# Insulin-induced localized lipoatrophy preceded by shingles (herpes zoster): a case report

**DOI:** 10.1186/1752-1947-8-223

**Published:** 2014-06-24

**Authors:** Ernst A Chantelau, Ruth Prätor, Jörg Prätor

**Affiliations:** 1 Practice of Endocrinology and Diabetology, PD Dr. Kimmerle, Aachener Strasse 196, 40223 Düsseldorf, Germany; 2 Cäcilienstr.40, 48431 Rheine, Germany

## Abstract

**Introduction:**

Localized involutional lipoatrophy of subcutaneous adipose tissue may develop due to subcutaneous injection of pharmaceutical preparations. The pathogenesis of this adverse drug reaction is unknown. The progression of localized involutional lipoatrophy ceases and occasionally it resolves after withdrawing the inducing agent. In case of localized involutional lipoatrophy due to subcutaneous insulin therapy, low-dose systemic corticosteroids may be curative despite ongoing insulin administration.

**Case presentation:**

We report a recurrence of insulin-induced localized involutional lipoatrophy at the abdominal wall in a 57-year-old Caucasian woman with type-1 diabetes on continuous subcutaneous insulin infusion. The first episode of insulin-induced localized involutional lipoatrophy two years previously had been cured by oral prednisone. The recurrence was treated immediately with 10mg prednisone once daily for five months, and was cured thereafter. The insulin analog preparation (Humalog™) and the insulin pump equipment (Accu-Chek Spirit™) applied were the same during both episodes. Both episodes were preceded by a temporary disturbance of the immune balance (the first episode by vaccination, the second episode through shingles).

**Conclusions:**

This case confirms that insulin-induced localized involutional lipoatrophy in type-1 diabetes can occur again, and can be cured by systemic corticosteroids. We suggest that temporary disturbance of the immune balance may trigger this transitory idiosyncratic reaction in a susceptible individual.

## Introduction

Localized involutional lipoatrophy (LIL) of subcutaneous adipose tissue is a rare condition, characterized by reduction of fat cells in size, and, occasionally, in number. The fat tissue involution develops without symptomatic inflammation, although histopathology of incipient cases may reveal few inflammatory cells among the atrophic fat tissue. Muscle tissue is never involved.

Lopez *et al.*[1] have obtained biopsies of lipoatrophies in three patients shortly after the last insulin injection; histopathology showed some lymphocytic, eosinophilic, and mast cell infiltration. However, direct immunofluorescence for immunoglobulin G (IgG), for immunoglobulin A (IgA), immunoglobulin M (IgM), complement component 3 (C3) and fibrin could not be demonstrated [1], in line with other authors [2]. Adipose tissue was atrophied, with focal fibrosis [1]. Adipocytes were shrunk and reduced in number, consistent with previous reports [3-5]. Milan *et al.*[6] provided ultrastructural analyses on chronic lipoatrophies, having existed for eight months to six years (median 26 months) in three patients using Humalog™. They showed fat cell atrophy, deposition of amyloid, and numerous perivascular preadipocytes. Quantitative polymerase chain reaction revealed a reduction in adipocyte-specific messenger ribonucleic acid (mRNA) (perilipin, adiponectin, fatty acid-binding protein 4, peroxisome proliferator-activated receptor gamma 2), whereas mRNA of adipocyte enhancer-binding protein 1 was enhanced, and of leptin was reduced to zero [6]. There was no evidence of inflammation. Rather, the histopathologic pattern was like that of fat tissue undergoing starvation, except for the amyloid deposition, that is conglomerates of denaturated exogenous insulin or insulin analog [7].

The etiopathogenesis of the LIL is unknown; it is associated with various immunological disorders (for example type-1 diabetes mellitus, immunogenic thyreopathies) and has a female preponderance. LIL is probably idiopathic, but develops predominantly after local mechanical trauma or subcutaneous administration of pharmaceuticals (for example insulin, antibiotics, vaccines, corticosteroids, growth hormone). The drug-induced type of LIL stops spreading after withdrawal of the agent. In children, insulin-induced LIL may resolve spontaneously after changing the insulin brand [8].

We have recently reported on a patient with incipient LIL induced by external insulin pump therapy with Humalog™ [5]. Within 10 months of low-dose oral prednisone treatment, the LIL resolved completely. We now report another episode of LIL in the same patient, at an insulin infusion site close to a previously lipoatrophic area.

## Case presentation

Our patient is a 57-year-old Caucasian woman born in 1956 and diagnosed with type-1 diabetes mellitus in 1969. At the time of writing this report, in March 2014, she is still free from significant diabetic retinopathy, nephropathy, and neuropathy. She has been treated with an insulin pump since 1984, and has been using a U-100 analog insulin lispro (Humalog™, Eli Lilly Deutschland, Bad Homburg, Germany) since 2004. The equipment applied from 2008 onward comprised an Accu-Chek Spirit™ insulin pump and Accu-Chek FlexLink™ infusion sets with 8mm steel cannula (all from Roche Diabetes Care, Burgdorf, Switzerland). The cannula part of the infusion set was changed every two days, and the tubing (60cm long) was changed every 10 days, together with the insulin reservoir inside the pump. The dose of Humalog™ was around 25 to 30 units/day. Apart from Humalog™, her current medication consists of levothyroxine 125μg once daily because of immunogenic thyroid atrophy. Her most recent glycated hemoglobin (HbA1c) test result (normal range 4 to 6 percent of total hemoglobin) was 6.3 percent, and her body mass index was 23 to 24kg/m^2^.

**Figure 1:**
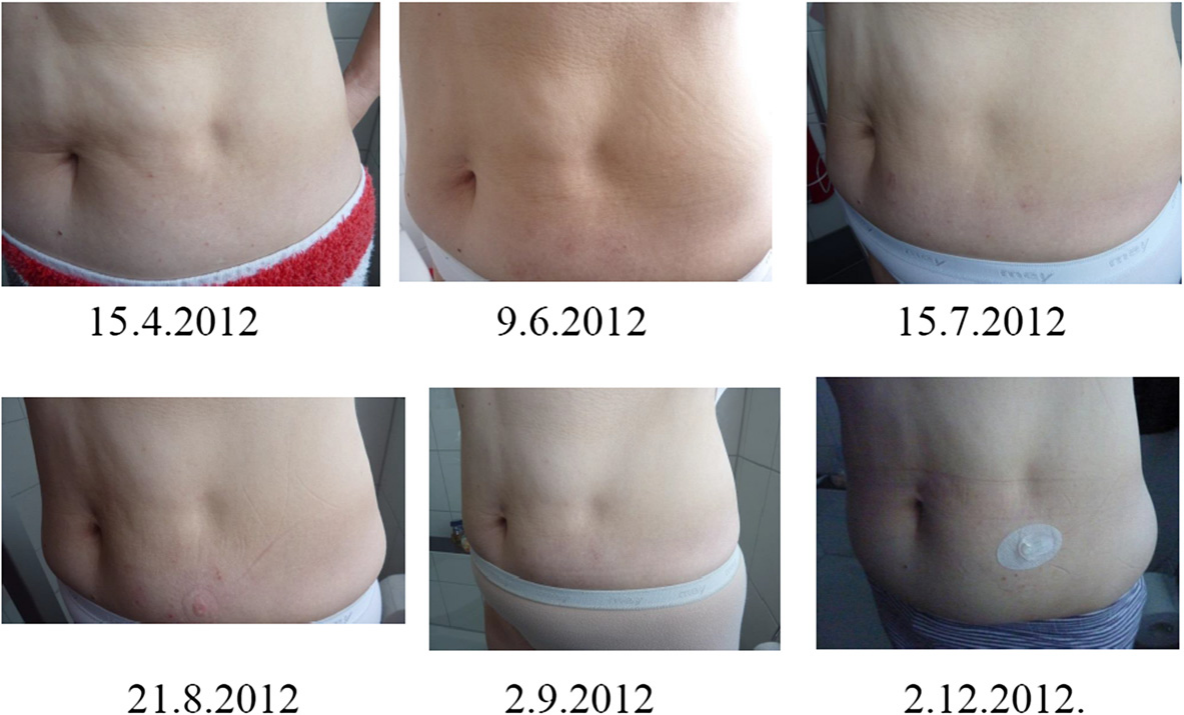
**Development of localized involutional lipoatrophy at the insulin infusion site before (15 April 2012) and during treatment with oral prednisone (10 mg/day from 15 April 2012 to 26 June 2012, 5 mg/day until 21 August 2012).** No prednisone from 22 August 2012 until December 2012.

Following a hepatitis vaccination with Twinrix™ (GlaxoSmithKline Dresden, Germany), in 2007 and 2008, our patient had experienced a first episode of LIL at the Humalog™ infusion sites on both sides of the umbilicus. The lipoatrophic defects were cured by low-dose oral prednisone therapy from 2009 to 2010, as we published in 2011 [5]. After a prolonged cold between December 2011 and January 2012, herpes zoster (shingles) broke out at the left thoracolumbar region (T 10 dermatome), and healed uneventfully in February 2012. Shortly thereafter, in March 2012, a solitary spot of subcutaneous fat atrophy was noticed at one catheter insertion site on the abdominal wall left of the umbilicus (Figure 1), and was diagnosed as LIL recurrence.

**Figure 2:**
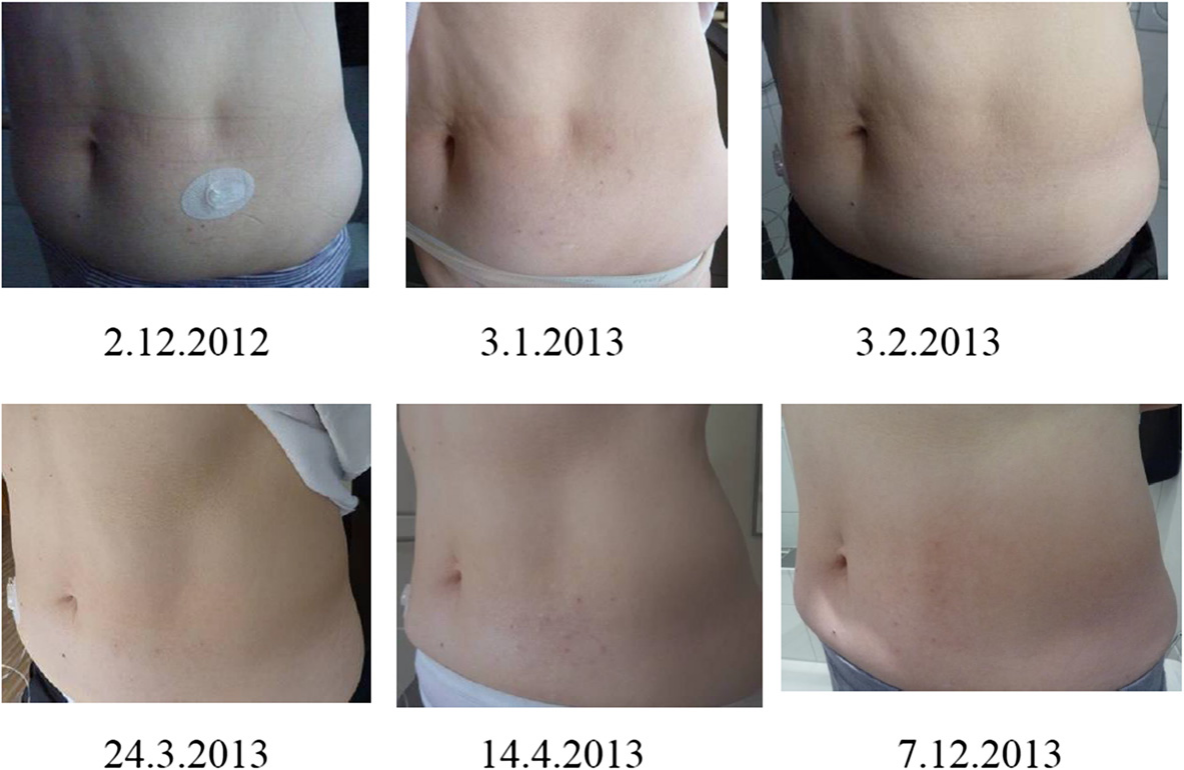
**Development of localized involutional lipoatrophy at the insulin infusion site, before (2 December 2012) and after resumption of 10mg prednisone daily (the end of December 2012 to mid-March 2013, tapered off until 24 March 2013).** No prednisone from 25 March 2013 until 31 December 2013.

Laboratory tests were limited to determinations of varicella-zoster virus (VZV) IgG-antibody titer (3230mIU/ml; enzyme-linked immunosorbent assay (ELISA), normal <149mIU/ml), VZV IgM negative; herpes simplex virus IgG-antibody titer (7 IU; normal <0.9 IU), human leukocyte antigen (HLA)-Cw7 (positive), HLA-Cw6 (negative), HLA-B27 (negative), HLA-DR4 (negative), and HLA-DRB1*03 allele (positive). Other markers of autoimmune diseases (like antinuclear antibodies (ANA), antinuclear cytoplasmatic antibodies (ANCA)) had previously been found negative [5]. Because of the limited information gained from biopsies during the first episode of LIL [5], biopsies were not repeated on this occasion.It was decided to carry on with the insulin preparation (Humalog™) and the pump equipment unchanged, at variance to the first LIL episode. Oral prednisone therapy was initiated by mid-April 2012, when the atrophic area was still relatively small (approximately 3cm in diameter, see Figure 1). The starting dose was 10mg/day, which was reduced deliberately to 5mg/day after nine weeks (the end of June 2012), and discontinued by the end of August 2012. The reasons for discontinuation were intolerable emotional disturbances and blood glucose instability, attributed to prednisone. At that time point, the lipoatrophy had improved (see Figure 1) but the excavation was not entirely refilled. There were no new lipoatrophic sites. By the end of December 2012, prednisone 10mg/day was resumed in order to improve the remaining lipoatrophy (Figure 2), and was tapered off by the end of March 2013, because the lipoatrophy was almost cured. The total duration of 10mg prednisone daily was 20 weeks (five months). In March 2014, the LIL was still in remission.

## Discussion

The present case confirms the effectiveness of 10mg prednisone orally once daily to cure an incipient insulin-induced LIL, localized at a Humalog™ infusion site, under conditions of ongoing subcutaneous Humalog™ application elsewhere at the abdomen. In contrast to a previous LIL episode in the same patient [5], prednisone treatment was required for only five months altogether, most likely because it was started early after the onset of the LIL when the defect was relatively small. The first episode of LIL had produced lipoatrophic defects which were more than twice as large (6×7cm) as the present one. They had existed already for up to 11 months before treatment was initiated and, hence, required prednisone for 10 months for complete resolution.

The etiopathogenesis of insulin-induced LIL is unknown. In our case, the mechanical needle trauma or the injected agent (Humalog™) potentially could have played a role, as both had been continued since the first LIL episode. The importance of the injected agent is emphasized by a previous placebo-controlled study of pegylated growth hormone [9]. While 13 percent of patients injecting pegylated growth hormone developed LIL, none of the patients injecting a placebo (containing the same components as the verum, except for the pegylated growth hormone, that is water, sodium phosphate, mannitol and glycine) did. The LIL resolved spontaneously within two to three months after cessation of the growth hormone preparation [9]. Likewise, insulin-induced LIL in a type-2 diabetic patient resolved spontaneously within six months after cessation of any insulin treatment [10].

In our patient it was not possible to stop the administration of insulin by subcutaneous route. Changing the insulin brand had proved ineffective previously [5]. Hence, we resorted to drug treatment by oral prednisone, as in the first LIL episode two years ago [5]. The effective dose was 10mg daily; 5mg/day was presumably not enough (see Figures 1, 2). Glucocorticoids have proved effective in other cases of incipient insulin-induced LIL [5,11-13]; however, the pathophysiological basis of the effectiveness remains to be determined. Is it the anti-inflammatory effect [14]? In our case, the incipient LIL did not appear inflamed (see Figures 1, 2). Is it the corticoid effect on fat cell differentiation? Hauner *et al.*[15] have demonstrated *in vitro* that glucocorticosteroids and insulin stimulate the differentiation of adipocytes from preadipocytes and their enlarging.

Similar to the first episode of LIL, which was preceded by a hepatitis vaccination, the second one was also preceded by an affection of the immune system, as evidenced by a herpes zoster rash four weeks before. Parallels may be drawn to infectious mononucleosis, an infection by another herpes virus (Epstein-Barr virus), which triggers an adverse drug reaction (exanthema) to ampicilline in susceptible patients [16,17]. Unfortunately, previous reports on insulin-induced LIL have never considered the possibility that certain intercurrent illnesses or disturbances of the immune system may have instigated the onset of the condition.

The signal for the drug reaction (fat tissue involution) in our case most likely originated from an interaction of the subcutaneously administered insulin lispro with some unknown transient immunologic (or other) idiosyncratic trigger. Idiosyncrasy, however, implies individual susceptibility [18]. Consistent with this theory, four other insulin-treated diabetic patients with herpes zoster, two of whom with significant thyroid autoimmunity [19], never exhibited LIL (see Table 1). They probably lack the specific susceptibility (E.A.C., unpublished).

**Table 1:** Clinical features of four diabetic patients with known history of herpes zoster, who never exhibited insulin-induced localized involutional lipoatrophy (LIL)

**Zoster patients without insulin-induced LIL**				
Gender	male	female	male	female
Age, years	56	60	59	79
Type of diabetes	type-1	type-1	type-1	type-2
Duration of diabetes, years	37	36	28	19
Diabetic complications^1^	none	none	none	none
Insulin therapy	injections	injections	CSII^2^	injections
Application of Humalog™	no	no	no	no
Varicella-zoster IgG, mU/ml^*^	1400	>2000	1400	>2000
Varicella-zoster IgM^*^	negative	negative	negative	negative
HbA1c, %	7.6	7.1	7.1	8.9
Postherpetic neuralgia	yes	yes	no	no
Other relevant features	yes^3^	yes^4^	yes^5^	yes^6^

^1^Neuropathy, retinopathy, nephropathy; ^2^continuous subcutaneous insulin infusion with external insulin pump; ^3^insulin lipohypertrophy: lipoma with focal fibrosis and amyloid deposition (histologically proven); ^4^thyroid autoantibody positive, ^5^immunogenic thyroid atrophy; ^6^multiple allergies; ^*^ELISA, enzyme-linked immunosorbent assay. CSII, continuous subcutaneous insulin infusion; IgG, immunoglobulin G; IgM, immunoglobulin M; HbA1c, glycated hemoglobin.

Various subcutaneously administered pharmaceuticals including hormone preparations [1-6,8-13,20,21] and vaccines [22] may be the cause of LIL. In cases of incipient insulin-induced LIL, the idiosyncratic trigger can obviously be turned off by systemic corticosteroid application, despite ongoing subcutaneous insulin administration. Moreover, corticosteroid treatment may help the atrophied fat tissue to regenerate. It remains to be demonstrated if corticosteroids are of similar effectiveness in other phases and types of LIL.

## Conclusions

The present case demonstrates the favorable outcome of insulin lispro (Humalog™) associated localized involutional lipoatrophy (LIL), which was treated early after its inception with low-dose prednisone. Of note, the LIL was a recurrence, and the present and the previous episode had been preceded by acute disturbances of the immune system. These observations lend support to the hypothesis that insulin-induced LIL may be a transitory idiosyncrasy.

## Consent

Written informed consent was obtained from the patient for publication of this case report and any accompanying images. A copy of the written consent is available for review by the Editor-in-Chief of this journal.

## Abbreviations

ANA: antinuclear antibodies; ANCA: antinuclear cytoplasmatic antibodies; C3: complement component 3; CSII: continuous subcutaneous insulin infusion; ELISA: enzyme-linked immunosorbent assay; HbA1c: glycated hemoglobin; HLA: human leukocyte antigen; IgA: immunoglobulin A; IgG: immunoglobulin G; IgM: immunoglobulin M; LIL: localized involutional lipoatrophy; mRNA: messenger ribonucleic acid; VZA: varicella-zoster virus.

## Competing interests

The authors declare that they have no conflict of interests.

## Authors’ contributions

EAC conceived the idea, compiled the data and drafted the manuscript. RP and JP contributed patient data and the photographs and co-authored the final version of the manuscript. All authors read and approved the final manuscript.
